# Effect of sodium hypochlorite solution and gel with/without passive ultrasonic irrigation on *Enterococcus faecalis*, *Escherichia coli* and their endotoxins

**DOI:** 10.12688/f1000research.24721.1

**Published:** 2020-06-24

**Authors:** Amjad Abu Hasna, Laiana Pereira Da Silva, Fernanda Carvalho Pelegrini, Cláudia Luísa Ribeiro Ferreira, Luciane Dias de Oliveira, Cláudio Antonio Talge Carvalho

**Affiliations:** 1 Department of Restorative Dentistry, Endodontic Division., São Paulo State University (UNESP), Institute of Science and Technology, São José dos Campos, São Paulo, 12245000, Brazil; 2 Department of Biosciences and Oral Diagnosis, São Paulo State University (UNESP), Institute of Science and Technology, São José dos Campos, São Paulo, 12245000, Brazil

**Keywords:** Sodium hypochlorite, Passive ultrasonic irrigation, Enterococcus faecalis, Escherichia coli, Endotoxins.

## Abstract

**Background: **Sodium hypochlorite (NaOCl) is the most commonly used irrigant in endodontics **. **The purpose of this study was to evaluate the effect of NaOCl solution (2.5%) and gel (3%) with/without passive ultrasonic irrigation (PUI) on *Enterococcus faecalis, Escherichia coli,* and their endotoxins, lipopolysaccharide (LPS) and lipoteichoic acid (LTA).

**Methods: **40 human lower premolars were contaminated with *E. coli *(ATCC 25922) for 28 days and *E. faecalis* (ATCC 29212) for 21 days. Specimens were randomly divided into four groups: (1) 2.5% NaOCl irrigating the canals without PUI activation; (2) 2.5% NaOCl with PUI; (3) 3% NaOCl gel irrigating the canals without PUI; and (4) 3% NaOCl gel with PUI. 40 mL of irrigant was used for each group. PUI activation was carried out using E1-Irrisonic stainless-steel tip at 10% frequency. After treatment, all specimens were filled with 3mL of 17% ethylenediaminetetraacetic acid (EDTA) for 3min and then washed with nonpyrogenic saline solution. Three samples were collected from the canals: S1, at baseline to confirm biofilm formation; S2 after treatment; and S3 after EDTA. Samples were assessed for *E. coli *and *E. faecalis *colony forming units, and LPS and LTA were assessed using chromogenic kinetic LAL assay and ELISA, respectively. Data were analyzed by Kruskal-Wallis, Friedmann and Dunn tests with α≤0.05.

**Results**: All groups were effective in reducing the microbial load of *E. coli* and *E. faecalis* after treatment without a significant difference among the groups. NaOCl and NaOCl gel groups had no significant difference in reducing LPS and LTA. Statistically increased reduction was seen for NaOCL + PUI and NaOCl gel + PUI compared for groups without PUI.

**Conclusions: **NaOCl gel has the same antimicrobial action of NaOCl solution and can partially detoxify endotoxins. PUI improves NaOCl (gel or solution) action over *E. faecalis *and *E. coli* and their endotoxins.

## Introduction

Sodium hypochlorite (NaOCl) is the most commonly used irrigant in endodontics ( Iqbal, 2012). It has been used since the second half of the 18 ^th^ century ( Sedgley, 2004) because it has antimicrobial action ( Luebke, 1967) and dissolves necrotic tissues ( Taylor & Austin, 1918).

*Enterococcus faecalis* is a Gram-positive bacterium found in the root canal system (RCS) and can be disinfected by NaOCl ( Siqueira *et al*., 2000). It may also be found in secondary infections of endodontically treated teeth ( Machado *et al*., 2020). In addition, *Escherichia coli,* a Gram-negative bacterium, is also found in endodontic infections ( Narayanan & Vaishnavi, 2010).

Bacteria have endotoxins in their outer membrane known as lipoteichoic acid (LTA) in Gram-positive bacteria ( Ginsburg, 2002) and lipopolysaccharide (LPS) in Gram-negative bacteria ( Mergenhagen & Varah, 1963). Endotoxins can be released during the duplication or death of these bacteria in infected RCS and this has a role in developing periapical lesions ( Endo *et al*., 2012). Endodontic treatment using NaOCl can detoxify endotoxins, but not completely ( Cavalli *et al*., 2017).

NaOCl is a cytotoxic substance ( Salazar-Mercado *et al*., 2019); overflow during endodontic treatment can cause diverse exacerbations ( Goswami *et al*., 2014). Thus, NaOCl gel may be safer due to its minor apical extrusion tendency ( Nesser & Bshara, 2019). Passive ultrasonic irrigation (PUI) improves RCS disinfection ( Plotino *et al*., 2007), removing the smear layer and vapor lock during endodontic treatment ( Bueno *et al*., 2019; Dioguardi *et al*., 2019), and permits greater penetration of irrigants to the dentinal tubules ( Sáinz-Pardo *et al*., 2014).

The purpose of this study was to evaluate the effect of NaOCl solution (2.5%) and gel (3%) with/without PUI on *E. faecalis, E. coli,* and their endotoxins, LTA and LPS, respectively.

## Methods

This study was approved by the research ethics committee of São Paulo State University, Institute of Science and Technology (n ^o^1.504.995). The teeth used in this study were obtained from clinics where teeth are donated during routine procedures and following authorization of the patients. The research team presented the terms of donation by the clinics from which the teeth where obtained to the research ethics committee when submitting the study methodology. A total of 40 human lower premolars were collected (based on dimensional and morphological similarities).

### Specimen preparation

To standardize root canal diameter, the teeth were initially instrumented with a #30 K-file (Maillefer, Ballaigues, Switzerland) and irrigated with 5 mL of NaOCl 1% for each file used. The canals were dried with sterile paper points (Dentsply Ind Com LTDA, RJ, Petrópolis, Brazil) and the apical region was sealed with light-cured resin composites (3M Dental Products, St Paul, MN). The outer surfaces of the specimens were covered with two layers of epoxy adhesive (Araldite - Brascola, Sao Paulo, Brazil), except the cervical opening ( de Oliveira *et al*., 2007). Then they were fixed with chemically activated acrylic resin in 24-well plates and sterilized by gamma radiation with cobalt 60 (20 KGy for 6 hours) ( Csako *et al*., 1983).

### Biofilm formation

Specimens were contaminated with *E. coli* (ATCC 25922) for 28 days and *E. faecalis* (ATCC 29212) for 21 days and incubated at 37°±1°C, following the protocol of Maekawa *et al.* (2013).

### Experimental groups

Specimens were divided into four experimental groups (n=10/group) as follows: (1) 2.5% NaOCl (Asfer, São Caetano do Sul, São Paulo, Brazil) irrigating the canals without PUI activation; (2) 2.5% NaOCl irrigating the canals with PUI; (3) 3% NaOCl gel (Ultradent, South Jordan, UT, USA) irrigating the canals without PUI; and (4) 3% NaOCl gel irrigating the canals with PUI. All specimens were instrumented as a part of the biomechanical preparation by Reciproc R40 (VDW, Munich, Germany) following the protocol of each experimental group ( Table 1).

**Table 1.** Protocols for experimental groups. NaOCL, sodium hypochlorite; PUI, passive ultrasonic irrigation.

Experimental group	Irrigation protocol (repeated three times in each third of the root canal)	Final wash
NaOCl	5 mL of NaOCl 2.5% during instrumentation without PUI and then 5 mL remained in the canal without any activation.	10 mL of 2.5% NaOCl solution without PUI activation.
NaOCl + PUI	5 mL of NaOCl 2.5% during instrumentation without PUI and then 5 mL activated with PUI	10 mL of 2.5% NaOCl solution activated with PUI.
NaOCl gel	Filled with 2 mL of 3% NaOCl gel and irrigated with 10 mL of saline solution during instrumentation without PUI.	Filled with 2 mL of 3% NaOCl gel and irrigated with 10 mL of saline solution without PUI activation.
NaOCl gel + PUI	Filled with 1 mL of 3% NaOCl gel and irrigated with 10mL of saline solution during instrumentation without PUI and then filled with 1 mL of 3% NaOCl gel and irrigated with 10mL of saline solution activated with PUI	Filled with 2 mL of 3% NaOCl gel and irrigated with 10 mL of saline solution activated with PUI.

PUI activation was performed using an E1-Irrisonic stainless-steel tip (Helse, Santa Rosa de Viterbo, Brazil) (0.10mm in diameter) at the working length using CVDente 100 ultrasound activator (CVDentus, São José dos Campos, Brazil) at 10% frequency.

After treatment, all specimens were filled with 17% ethylenediaminetetraacetic acid (EDTA) (Biodinâmica, Ibiporã, PR, Brazil) for 3min and then washed with nonpyrogenic saline solution.

### Sample collection

Three samples were collected during the experiment, as in ( Maekawa *et al*., 2013): S1, at baseline to confirm biofilm formation; S2, immediately after treatment; and S3, after EDTA application.

### Colony forming unit (CFU/mL)

Serial dilutions of all samples were performed with sterile saline solution and aliquots of 30µl of each sample were seeded in two different culture medias: Enterococcosel agar (Becton, Dickinson and Company Sparks, MD, USA) for *E. faecalis;* and MacConkey agar (Himedia Laboratories, Mumbai, India) for *E. coli*. The plates were kept at 37°C for 24h and then CFU/mL were counted.

### Quantification of LPS/LTA levels

LPS levels in each sample was assessed as in Machado *et al*. (2020) using kinetic chromogenic limulus amebocyte lysate assay (Lonza, Walkersville, MD, USA). The plates were incubated at 37±1°C for 10 min in a KineticQCL reader, which was coupled to a computer with the WinKQCL software (Lonza). As soon as the kinetic test started, absorbance at 405 nm was read in each microplate well and automatically calculated the log/log linear correlation between reaction time of each standard solution and corresponding endotoxin concentration.

LTA was assessed using enzyme-linked immunosorbent assay using ELISA 96-well plates (Nunc Thermo Scientific, Waltham, MA, USA) sensitized with anti-LTA monoclonal antibody (manufacturer) and kept overnight at relative humidity. Next day, the plates were washed with a wash buffer (PBS with 0.05% Tween 20) and incubated with a blocking buffer (PBS with 2% bovine serum albumin, BSA) for 1 h at room temperature. Then, they were washed with a wash buffer and received 100 μL of the samples collected and 100 μL of the LTA standard followed by serial 2-fold dilutions (standard curve) and maintained for 2 hours at room temperature. Afterwards, the plates were washed again and 100 µL of anti-LTA antibody was added for 1 hour at room temperature. The plates were washed again and 100 μl of horseradish peroxidase HRP conjugated rabbit IgG antibody was added for 1 hour at room temperature. Lastly, the plates were washed, and the reaction was developed using tetramethylbenzidine (TMB). After 20 min under the light, 50 μL of stop solution (2 N sulfuric acid) was added to each well of the plate and optical densities were read in the microtiter plate reader (BioTek Instruments, Inc., Winooski, VT, USA) at 450 nm absorbance ( Machado *et al*., 2020).

### Statistical analysis

Data were analyzed using Kruskal-Wallis, Friedmann and Dunn tests with α≤0.05 byGraphPad Prism 6 (La Jolla, CA, USA)

## Results

**Figure 1.**
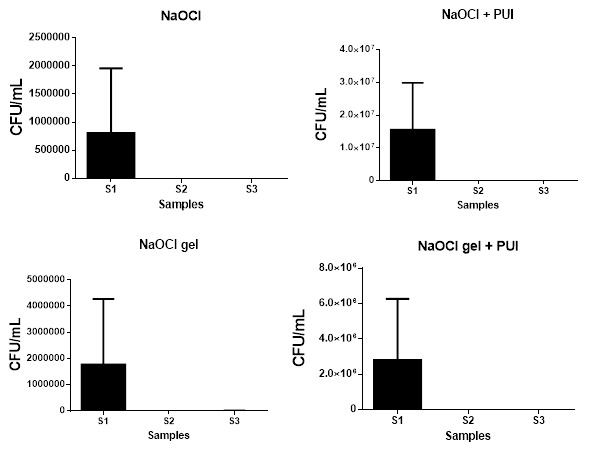
Statistical difference among the samples of each experimental group for *E. coli* (CFU/mL). CFU, colony forming units; NaOCL, sodium hypochlorite; PUI, passive ultrasonic irrigation.

**Figure 2.**
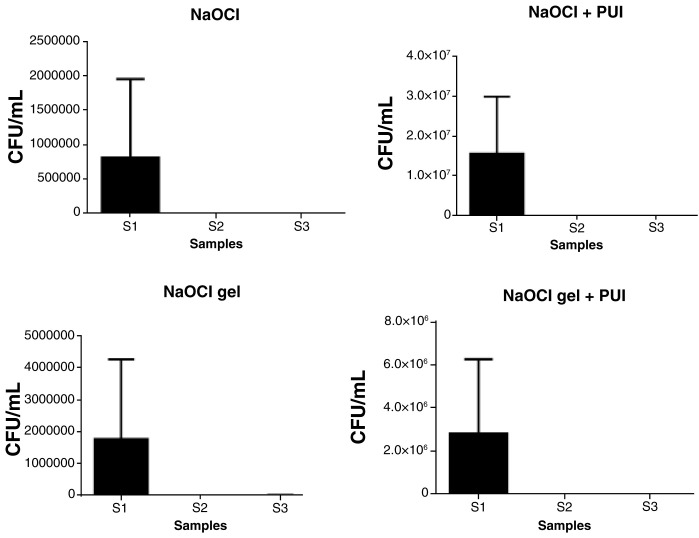
Statistical difference among the samples of each experimental group in *E. faecalis* (CFU/mL). CFU, colony forming units; NaOCL, sodium hypochlorite; PUI, passive ultrasonic irrigation.

All experimental groups were effective in reducing the microbial load (CFU/mL) of *E. coli* ( Figure 1) and *E. faecalis* ( Figure 2) in S2 (from S1 levels). There was no significant difference among the experimental groups for S2 or S3 ( Table 2).

**Figure 3.**
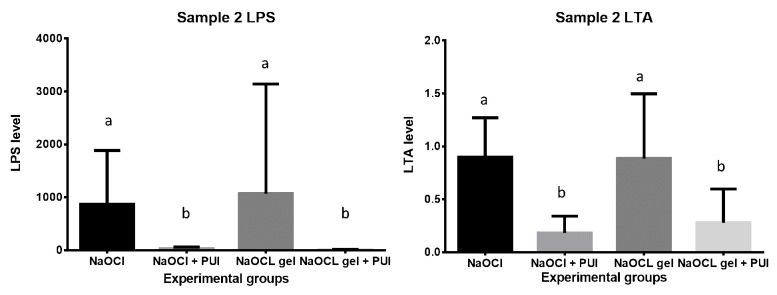
Statistical difference among the experimental groups at S2 for LPS and LTA levels. NaOCL, sodium hypochlorite; PUI, passive ultrasonic irrigation; LPS, lipopolysaccharide; LTA, lipoteichoic acid.

NaOCl and NaOCl gel groups had no significant difference in reducing LPS and LTA at S2. Groups with PUI showed a statistically increased reduction in LPS and LTA compared with groups without PUI ( Figure 3). As a limitation of this study, the LPS and LTA levels were not assessed in S3.

**Table 2.** Median of colony forming units/mL for *E. coli* and *E. faecalis* in all samples (S1, S2 and S3). NaOCL, sodium hypochlorite; PUI, passive ultrasonic irrigation.

	*E. coli*			*E. faecalis*		
Samples	S1	S2	S3	S1	S2	S3
NaOCl	30x10 ^4^ (366x10 ^4^- 10x10 ^4^) A-a	0 (0-0) B-a	0 (0-0) B-a	314.95x10 ^4^ (68x10 ^5^- 56.6x10 ^4^) A-a	0 (0-0) B-a	0 (0-0) B-a
NaOCl + PUI	983x10 ^4^ (29x10 ^6^- 3x10 ^6^) A-b	0 (0-0) B-a	0 (0-0) B-a	585x10 ^4^ (79x10 ^5^-26.6x10 ^4^) A-a	0 (0-0) B-a	0 (0-0) B-a
NaOCl gel	45x10 ^4^ (756x10 ^4^- 1x10 ^4^) A-a	0 (0-0) B-a	0 (0-33667) B-a	218x10 ^4^ (686x10 ^4^- 1x10 ^4^) A-a	0 (0-333) B-a	0 (0-0) B-a
NaOCl gel + PUI	88333 (880x10 ^4^-33x10 ^6^) A-a	0 (0-0) B-a	0 (0-0) B-a	485x10 ^4^ (71x10 ^5^- 18x10 ^5^) A-a	0 (0-333) B-a	0 (0-0) B-a

*Different letters indicate statistically significant differences (p<0.05). Uppercase letters indicate difference in rows (Friedman test; intra-groups) and lowercase letters indicate difference in columns (Kruskal-Wallis test; inter-groups).

## Discussion

Well endodontically treated teeth fail mainly because of secondary intraradicular infection ( Siqueira, 2001). *E. faecalis* and *E. coli,* among other bacteria, are the most detected microorganisms in periapical lesions ( Geibel *et al*., 2005).

The use of NaOCl as an irrigant cannot be over-emphasized. In this study, it was effective in disinfecting *E. faecalis* and *E. coli* ( Table 2). These results agree with the results of Siqueira *et al*. (2000), who showed that NaOCl was an effective irrigant over *E. faecalis* at different concentrations (1%, 2.5%, and 5.25%). In addition, Valera *et al*. (2009) showed that 1% NaOCl is effective in disinfecting *E. faecalis* inoculated in the RCS. There have also been more recent studies that have the same results ( Carvalho *et al*., 2020; Plutzer *et al*., 2018; Valera *et al*., 2014). Similarly for *E. coli*, NaOCl has been reported in the literature as an effective irrigant in disinfecting microorganisms in the RCS lumen at 2.5% concentration ( Maekawa *et al*., 2011) and in preventing *E. coli* regrowth ( Valera *et al*., 2015).

In the present study, NaOCl was not effective in detoxifying LPS and LTA completely ( Figure 3). In support of this, the literature has shown that NaOCl is not effective in reducing endotoxin levels in the RCS ( de Oliveira *et al*., 2007), i.e. it reduces the endotoxin level but does not completely eliminate them ( Neelakantan *et al*., 2019). However, adding chloride alkali electrolyte-stable anionic surfactant has been shown to improve NaOCl effectivity because it reduces superficial tension ( Valera *et al*., 2015).

NaOCl gel has been suggested as an alternative endodontic irrigant because theoretically it has the same antimicrobial action of NaOCl solution, but with less apical extrusion and could thus be safer ( Nesser & Bshara, 2019). It is effective in reducing the microbial load, but has been shown to be less effective when compared to NaOCl solution of a lower concentration ( Poggio *et al*., 2010; Zand *et al*., 2016). In the present study, NaOCl gel was shown to be just as effective as NaOCl solution in reducing microbial load.

To the best of our knowledge, there are no studies in the literature evaluating the effect of NaOCl gel over endotoxins. In this study it was statistically as effective as the solution. But both were more effective when combined with PUI as it increases NaOCl penetration into dentinal tubules ( Faria *et al*., 2019). The present study is novel as there are no studies evaluating how PUI can affect NaOCl solution or gel action on endotoxins.

PUI is still being studied due to divergence of results in the literature. For example, Paiva *et al.* (2013) used PUI after instrumentation and showed it was ineffective in reducing microbial load; however, Mohmmed *et al.* (2018) showed that PUI is effective in biofilm removal from lateral canals. However, this activity may be influenced by the irrigation protocol (irrigation time; irrigant volume; instrument shape and material; and the irrigation frequency and intensity) ( van der Sluis *et al*., 2007).

In conclusion, our study showed that NaOCl gel has the same antimicrobial action of NaOCl solution and can partially detoxify endotoxins. PUI improves NaOCl (gel or solution) action over *E. faecalis* and *E. coli* formation and their endotoxins (LPS and LTA).

## Data availability

### Underlying data

Harvard Dataverse: Raw Data of NaOCl solution and gel, https://doi.org/10.7910/DVN/JNK3TH ( Abu Hasna, 2020).

Data are available under the terms of the Creative Commons Zero "No rights reserved" data waiver (CC0 1.0 Public domain dedication).
