# Supplementary material for: Effect of sodium hypochlorite solution and gel with/without passive ultrasonic irrigation on Enterococcus faecalis, Escherichia coli and their endotoxins
Source: F1000Res. 2020 Jun 24;9:642. [Version 1] doi: 10.12688/f1000research.24721.1 (PMC7573735; doi:10.12688/f1000research.24721.1)
Supplement: Peer review history

# Peer review: Reviewer response for version 1

**DOI:** 10.5256/f1000research.27270.r69901

**Published:** 2020-10-19

**Authors:** Brenda P. F. A. Gomes

**Affiliations:** 1 Department of Restorative Dentistry, Division of Endodontics, Piracicaba Dental School, State University of Campinas‐UNICAMP, Piracicaba, Brazil

The manuscript evaluates the effect of NaOCl solution (2.5%) and gel (3%) with/without passive ultrasonic irrigation (PUI) on Enterococcus faecalis, Escherichia coli, and their endotoxins, lipopolysaccharide (LPS) and lipoteichoic acid (LTA). The article is very well written, responding adequately to the objectives proposed in this study. The language should be revised. The present study has a great clinical relevance.

 The title of the work is clear, concise, short and objective. Furthermore, it summarizes the authors conclusions, which is a positive point.

 The abstract proposed by the authors clearly summarizes the objective, materials and methods, statistical analysis, results and conclusions.

 The introduction presents a background based on previous studies and at most recent literature. The authors could add a paragraph about the use of PUI in endodontics to address all of the research factors.

 The authors did not report power calculation of the sample. Was the sample size based on previous studies? They should answer this question in the text.

 The authors well described the specimens’ preparation, the tests performed and the statistical analyses used in the study.

 The results section is well described and synthetizes the results obtained in the present study. The tables and figures are descriptive. Table 2 will be better viewed if it is before the discussion and the results present in this table could be better explored in the written presentation of the results.

 In the discussion, the authors could add one more paragraph about the use of PUI, as suggested in the introduction.

 The conclusions of this paper drawn adequately supported by the results.

Is the work clearly and accurately presented and does it cite the current literature?

Yes

If applicable, is the statistical analysis and its interpretation appropriate?

Yes

Are all the source data underlying the results available to ensure full reproducibility?

Yes

Is the study design appropriate and is the work technically sound?

Yes

Are the conclusions drawn adequately supported by the results?

Yes

Are sufficient details of methods and analysis provided to allow replication by others?

Yes

Reviewer Expertise:

Endodontics

I confirm that I have read this submission and believe that I have an appropriate level of expertise to confirm that it is of an acceptable scientific standard.

# Peer review: Reviewer response for version 1

**DOI:** 10.5256/f1000research.27270.r65501

**Published:** 2020-07-22

**Authors:** Talal Al-Nahlawi

**Affiliations:** 1 Department of Endodontics and Operative Dentistry, Syrian Private University, Damascus, Syria

This is a well designed study that evaluated the effect of sodium hypochlorite as an irrigant solution and gel over one Gram-positive bacterium and another Gram-negative one, as well over their endotoxins with and without passive ultrasonic irrigation (PUI). 

 This study may be the first study that evaluated the effect of sodium hypochlorite gel over the endotoxins of endodontic pathogens and this has a great clinical relevance.

 The introduction:

 It presented briefly the current literature and a historic background about the use of sodium hypochlorite. As a suggestion for the researchers in their future projects, I think that it is more appropriate to test another Gram-negative bacterium than e.coli. Even e.coli was found in the root canal system, however, I think that there are a variety of microorganisms that may be tested and have more relevance in the literature as example: Porphyromonas gingivalis (P. gingivalis) as in the study of Wang et al (2019) ^1^ and others in the study of Lukic et al (2020) ^2^.

 The methods:

 The study design was carefully planned, I think that the study in its current version is accepted to be indexed, all the cited articles in the methodology section have more details about the execution of this study. I am just wondering why the researchers did not use the sodium hypochlorite in the same concentration in both solution and gel forms? It is not a big deal here as the concentration is almost the same (2.5 and 3 %), however, I think this should be explained in the discussion section, or at least mentioning a previous study that used different concentrations. 

 The discussion:

 Why did the authors not take advantage of the positive results obtained from using PUI in this study to be discussed furtherly in the discussion section or even in the introduction section? I think another paragraph will improve the discussion section. The conclusions of this paper drawn adequately supported by the results.

Is the work clearly and accurately presented and does it cite the current literature?

Yes

If applicable, is the statistical analysis and its interpretation appropriate?

Yes

Are all the source data underlying the results available to ensure full reproducibility?

Yes

Is the study design appropriate and is the work technically sound?

Yes

Are the conclusions drawn adequately supported by the results?

Yes

Are sufficient details of methods and analysis provided to allow replication by others?

Yes

Reviewer Expertise:

Endodontics and operative dentistry

I confirm that I have read this submission and believe that I have an appropriate level of expertise to confirm that it is of an acceptable scientific standard.

## References

[1] : ZnO nanoparticles inhibit the activity of Porphyromonas gingivalis and Actinomyces naeslundii and promote the mineralization of the cementum. *BMC Oral Health*.2019;19(1) : 10.1186/s12903-019-0780-y 10.1186/s12903-019-0780-y 31088450PMC6518662

[2] : Endodontic-Like Oral Biofilms as Models for Multispecies Interactions in Endodontic Diseases. *Microorganisms*.2020;8(5) : 10.3390/microorganisms8050674 10.3390/microorganisms8050674 32384777PMC7285038
